# Not all effort is equal: the role of the anterior cingulate cortex in different forms of effort-reward decisions

**DOI:** 10.3389/fnbeh.2014.00012

**Published:** 2014-01-28

**Authors:** Victoria Holec, Heather L. Pirot, David R. Euston

**Affiliations:** Department of Neuroscience, Canadian Centre for Behavioural Neuroscience, University of LethbridgeLethbridge, AB, Canada

**Keywords:** anterior cingulate, effort, decision making, courage

## Abstract

The rat anterior cingulate cortex (ACC) mediates effort-based decision making when the task requires the physical effort of climbing a ramp. Normal rats will readily climb a barrier leading to high reward whereas rats with ACC lesions will opt instead for an easily obtained small reward. The present study explored whether the role of ACC in cost-benefit decisions extends beyond climbing by testing its role in ramp climbing as well as two novel cost-benefit decision tasks, one involving the physical effort of lifting weights and the other the emotional cost of overcoming fear (i.e., “courage”). As expected, rats with extensive ACC lesions tested on a ramp-climbing task were less likely to choose a high-reward/high-effort arm than sham controls. However, during the first few trials, lesioned rats were as likely as controls to initially turn into the high-reward arm (HRA) but far less likely to actually climb the barrier, suggesting that the role of the ACC is not in deciding which course of action to pursue, but rather in maintaining a course of action in the face of countervailing forces. In the effort-reward decision task involving weight lifting, some lesion animals behaved like controls while others avoided the HRA. However, the results were not statistically significant and a follow-up study using incremental increasing effort failed to show any difference between lesion and control groups. The results suggest that the ACC is not needed for effort-reward decisions involving weight lifting but may affect motor abilities. Finally, a courage task explored the willingness of rats to overcome the fear of crossing an open, exposed arm to obtain a high reward. Both sham and ACC-lesioned animals exhibited equal tendencies to enter the open arm. However, whereas sham animals gradually improved on the task, ACC-lesioned rats did not. Taken together, the results suggest that the role of the ACC in effort-reward decisions may be limited to certain tasks.

## Introduction

The precise role of the anterior cingulate cortex (ACC) remains unclear, but not for lack of ideas. The ACC has been implicated in error detection (Holroyd and Coles, 2002), conflict monitoring (Botvinick et al., 2001), self-directed action (Passingham et al., 2010), representation of action values (Rushworth, 2008), the subjective experience of pain (Shackman et al., 2011), and both recent and remote memory (Euston et al., 2012). The ACC has also been implicated in guiding effort-reward decision making. The primary impetus for this view comes from a series of studies that show that rats with ACC lesions are less willing to climb a wire mesh barrier to reach a high reward, opting instead to pursue a smaller but easier to reach reward (Walton et al., 2002, 2003; Rudebeck et al., 2006). These and other rodent studies have been followed by fMRI studies in humans, which have shown that the dorsal ACC encodes the value of an offered reward discounted by the effort needed to achieve it (Croxson et al., 2009; Prevost et al., 2010). Other studies suggest that ACC simply encodes the amount of actual or anticipated effort (Botvinick et al., 2009; Kurniawan et al., 2013). Single-cell recordings in monkeys also show that the dorsal ACC encodes both reward and anticipated effort (Kennerley et al., 2009) although recent finding suggest this may be specific to certain training conditions (Hosokawa et al., 2013). Combined with other studies, the results suggest that the ACC is part of a network of regions, including the amygdala, ventral striatum, and midbrain dopaminergic circuits, which play a crucial role in “weighing up the benefits of work” (Walton et al., 2006; Floresco et al., 2008).

Based on the preceding studies, one might suppose that the ACC would be necessary for all forms of cost-benefit decisions but, in fact, the evidence suggests its role is limited to only certain forms of cost. The original finding was obtained using a T-shaped maze, developed earlier by Salamone et al. (1994), in which a wire-mesh barrier is placed in the arm leading to high reward, while no barrier is present in the low-reward arm (LRA). The finding that rats with ACC damage or inactivation will forgo the high-effort/high-reward arm (HRA) has been replicated many times with only one contrary finding in a recent study with mice (Walton et al., 2003; Schweimer and Hauber, 2005; Rudebeck et al., 2006; Floresco and Ghods-Sharifi, 2007; Hauber and Sommer, 2009; Solinsky and Kirby, 2013). In this task, the activity of single ACC cells appears to be selective for the economically advantageous arm, further supporting the role of the ACC in tasks involving climbing barriers (Hillman and Bilkey, 2010). A recent electrophysiological study also suggests that the ACC is involved in reward-related decisions where the cost involves social competition (Hillman and Bilkey, 2012). On the other hand, attempts to demonstrate a role for the ACC in effortful tasks involving multiple lever presses (i.e., an instrumental ratio schedule) have yielded mixed results. In one case, rats with ACC lesions were less likely to choose a lever requiring a high response ratio and instead opted for a low-response ratio lever yielding low reward (Walton et al., 2009). However, in another study in which the lever-press ratio was gradually increased, rats with ACC lesions showed the same break point as normal rats (Schweimer and Hauber, 2005). When effort involves delaying a response to receive a reward, as in studies of intertemporal choice or delay discounting, the ACC is not required, but other areas such as the nucleus accumbens and orbitofrontal cortex are (Cardinal et al., 2001; Rudebeck et al., 2006). In sum, the ACC is not needed when effort involves waiting, and even when the discussion is limited to physical effort, there is some question as to the generality of ACC's role.

One area of cost-benefit decision making that has not received as much attention involves the pursuit of goals associated with fear or anxiety. In this case, the subject has to exert mental effort to overcome fear, a form of effort which we operationally defined here as “courage.” In a recent study with monkeys, subjects had to choose between a high reward associated with an aversive airpuff and a low reward without airpuff (Amemori and Graybiel, 2012). The dorsal ACC had separate groups of cells encoding either the positive or negative subjective value of each choice. Further, in one region with an abundance of cells encoding negative values, microstimulation caused subjects to avoid punishment. In humans, Nili et al. (2010) have shown that the fMRI BOLD signal in sub-genual ACC is stronger when subjects decide to bring a feared snake closer to their body as opposed to pushing it further away. This area is anatomically distinct from the dorsal ACC loci identified in other effort-reward tasks, raising the interesting possibility that different divisions of ACC are recruited for effort and courage tasks. In rodents, the role of ACC in courage-reward decisions has not previously been studied. However, it is known that lesions to adjacent medial prefrontal cortex have, in general, an anxiolytic effect in tests of unconditioned fear (Lacroix et al., 2000; Deacon et al., 2003; Shah and Treit, 2003). Lesions limited to ACC, however, apparently do not affect anxiety (Rudebeck et al., 2007). Hence, when faced with a situation requiring courage to reach a high reward, rats with ACC lesions might be expected to behave in one of two ways. If the ACC supports the deployment of courage as suggested by Nili et al. (2010), then one might suppose that rats with an ACC lesion would be less willing to display courage to achieve higher reward. If, on the other hand, ACC lesions have an anxiolytic effect, then animals might be *more* likely to approach a fearful situation after ACC lesions.

In the present experiment, we sought to test the specificity of ACC for different types of cost-benefit decisions. To ensure that our results were comparable to the previous literature, we replicated the ramp-climbing experiments of Walton et al. (2002) and others using similar ACC lesions. We also tested the role of the ACC in two novel cost-benefit tasks, one involving another form of physical effort and the other involving courage. In the physical effort task, rats were presented with a choice of two operant levers that required different amounts of force to depress to receive reward. The high-force lever yielded larger reward than the low-force lever. As this task was directly analogous to the ramp-climbing studies, we expected that rats with ACC lesions would forgo the high-effort/high-reward lever and opt instead for the low-effort/low-reward lever. For the courage task, rats were tested in a Y-shaped maze with a choice between a HRA that required traversal of an exposed wire mesh trellis and a LRA that was enclosed with high walls. If the role of ACC in cost-benefit decisions were to generalize to courage effort, then rats with ACC lesion should be less likely to enter the high-reward, high-courage arm. However, as discussed above, there were reasons to suspect that lesions might make animals less fearful, meaning that they would be more likely to enter the HRA.

## Materials and methods

### Experimental overview

**Figure 1:**
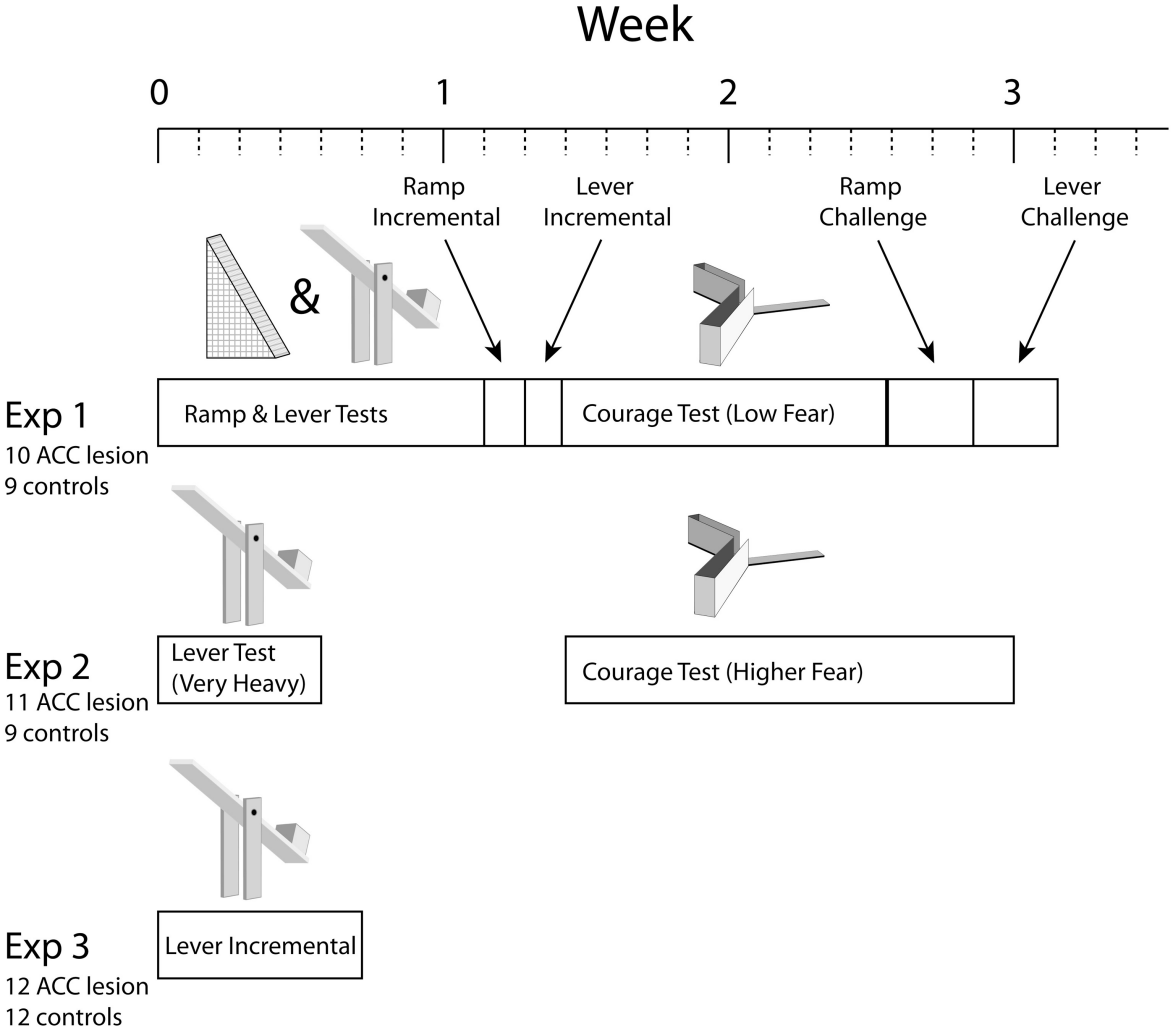
**Overview of experimental timelines**. Note that all experiments started with 24–26 animals but some animals were excluded due to surgical complications or misplaced lesions. Numbers shown were the totals after all such exclusions. Individual tests also had performance criteria which reduced numbers further in some cases. Please see Methods for further explanation.

As illustrated in Figure 1, three separate experiments were conducted, each performed on a separate group of rats and consisting of a different series of tests. Each test involved a choice between some form of cost associated with a high amount of food reward or minimal cost associated with low reward. Three basic types of cost were used: ramp climbing, weighted lever pressing, and courage. Note that rats were pre-trained in ramp and/or lever tasks for several weeks before surgery (not shown in the figure). Importantly, in each test, rats had to achieve specific behavior criterion during pre-training to be included in analysis. Because some rats achieved criterion on some tasks but not others, the number of rats in each analysis is different.

In Experiment 1, rats were trained on both the ramp and weight-lifting tasks simultaneously (ramp in the morning, weighted levers in the afternoon) before surgery and tested in a similar manner after surgery. Testing in both ramp and lever tasks involved 2–3 days of choices between high effort/high reward vs. low effort/low reward followed by 1 day in which rewards remained fixed but effort was high on both arms (the “equate effort” test). Rats were then tested in an incremental version of the ramp task in which the ramp height was increased every 10 trials within a session to generate an effort discounting curve. Next, a parallel incremental task was run in the lever apparatus. Following this, rats were trained and then tested in the courage task. Continuing with this series of tests, rats were run in a “ramp challenge” task in which the rat was re-trained on the high/low arm choice without any ramps and then tested the following day with an extremely high ramp (50.8 cm). This task was meant to test whether ACC lesions lead to specific impairment in the adjustment to sudden changes in task conditions. A similar challenge test was then conducted with weighted levers. In the final test of Experiment 1, rats were observed in the open field test (not shown in Figure 1).

The results of Experiment 1 suggested that the weights used in the lever task were not sufficiently heavy to deter any rats from the HRA. Similarly, results from the courage task suggested that entering the exposed arm was not that fear-provoking. Consequently, in Experiment 2 a new group of lesion and control rats was tested on the weight-lift task again but using a much heavier weight (40% of their body weight compared with 25% in Experiment 1). Following this, the courage task was run a second time using a higher amount of ambient illumination, hence increasing the sense of exposure on the open arm. As in the first experiment, rats were then tested in the open field test (not shown).

In the weight-lift test in Experiment 2, many rats could not reach our criterion of 80% HRA choices before surgery. Hence, Experiment 3 was conducted to re-examine whether the ACC is, in fact, necessary for effort-reward decisions involving weighted levers. In this test, pre-surgical training exposed the rats to unweighted levers yielding low and high reward but purposely excluded experience lifting weights. Hence, this test examined the response to de novo effort, offering what we felt were the most likely conditions to expose a difference between lesion and control animals.

### Animals

A total of 74 male Long–Evans rats (Charles River Laboratories International Inc., Montreal, QC) were used in three experiments in this study. Rats were 3 months of age at the start of training and maintained at 85% of their free-feeding weight (350–450 g) for the duration of training and testing. For at least 1 day before and 5 days after surgery, rats were allowed *ad libitum* food. In Experiment 1 started with 24 animals of which three died from peri-surgery complications, two sham animals were excluded from data analysis because of accidental brain damage to the region of interest, and one animal did not complete open field testing due to unrelated health problems. In Experiment 2, a total of 26 animals were used but three animals died from peri-surgery complications, two animals were removed from the study due to health problems and one sham animal was excluded from all analyses because of accidental brain damage. In Experiment 3, a total of 24 animals were used and no animals were excluded. Rats were maintained on a 12-h light-dark cycle and tested during the dark phase. All procedures were in accordance with the University of Lethbridge institutional animal care and use committee and Canadian Council on Animal Care recommendations and guidelines.

### Apparatus and task procedures

#### Food delivery

Animals received a high-calorie, chocolate-flavored liquid as reward (Ensure®, Abbott Laboratories, Brockville, ON) on all tasks. Food delivery was gravity driven though a silicone tube (91 cm long; inner diameter 1.98 mm, outer diameter 3.18 mm, wall thickness 0.61 mm; VWR International, Mississauga, ON) and controlled by pinch valves (Model SCH284B004-12/DC, ASCO Scientific, Florham Park, NJ). For each experiment, three 60 mL syringes served as food reservoirs and were hung 51 cm from the top of the track. Food was delivered into a 24 cm diameter conical food well via a hole in the floorboard, keeping the delivery tube inaccessible to the animal.

#### Automated experiment control

Control of all experimental events and data logging was supported by a standard computer running Microsoft Windows using a programmable digital input/output board (National Instruments PCIe-7841R, Toronto, ON) and custom software written in Microsoft Visual Basic and/or Labview (National Instruments, Toronto, ON).

#### Ramp-climbing

**Figure 2:**
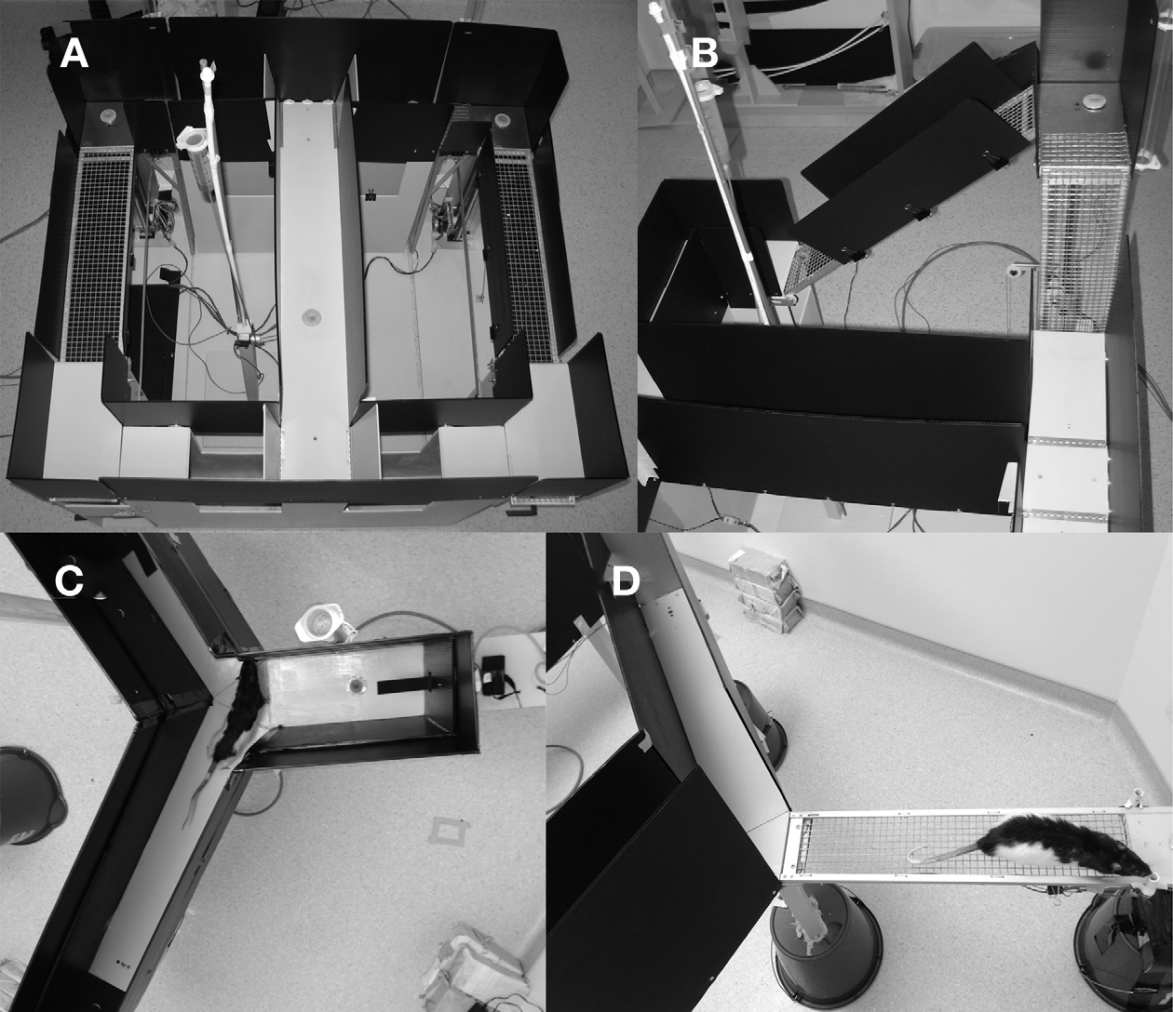
**Pictures of the three mazes used in these experiments. (A)** The ramp maze shown from the top with all gates in the up position. Reward platforms are in upper left and right and small circles are feeder wells. Both ramps are at their lowest position. **(B)** Close up of the left ramp in an elevated position showing the vertical ramp that rats climbed to reach reward and sloped return ramp. **(C)** Top view of the weighted lever apparatus showing a rat coming from the base arm and deciding which lever arm to choose. The dark bar is the lever and the small circle near the lever end is the feeder well. **(D)** A rat on the exposed arm of the courage maze. Reward well is located at the far right, just beyond the field of view. The enclosed safe arm is also visible.

An automated Figure 8 shaped maze was used for this task, an adaptation of the standard T-shaped ramp maze most often used in the literature (Salamone et al., 1994; Walton et al., 2002) to form a continuous maze (Figure 2A). The maze, made of wood and painted gray, measured 102 cm long by 114 cm wide and was elevated 60 cm from the floor. The running surface itself was 15 cm wide and was enclosed on both sides by black plastic walls, 36 cm in height. Feeders were located on the stem of the T and at the end of each choice arm. Both choice arm feeders were located on a 6 cm by 15 cm platform attached to a carriage which slid on a guide rail and could be raised from 0 to 51 cm above the surface of the maze. The platform had wire mesh down one side, presenting a vertical wall that the rat could climb to reach the reward (Figure 2B). Wire mesh was made of 1.6 mm thick galvanized steel wire with a 1.25 cm square spacing. Ramp height was varied via a rack and pinion gear system using stepper motors (Model 23Y9, Anaheim Automation, Anaheim, CA) driven by a stepper motor controller (Model G251X, Gecko Drive, Tustin, CA). The platform was connected to the return arm via a sloped wire-mesh ramp whose angle varied with the height of the platform. Finally, automated gates were used to control the rat's running direction, two located at the entry to the stem of the T and two at the exit (Figure 2A).

The task was conducted in a dimly lit room illuminated by 12 small white LEDs located around the ceiling-mounted camera. Animals wore a flexible belt made of Coban (3M, St. Paul, MN) to which red reflective tape was affixed for video tracking. Video tracking was provided via a standard video camera by Cheetah neurophysiology data acquisition software (Neuralynx, Bozeman, MT) running on a separate computer. The rat's position was communicated to the control computer in real-time, so that predefined spatial zones could trigger responses based on the animal's location on the track.

Rats were always started on the stem of the original T-figure, where two automated gates prevented them from entering the return arms. After receiving reward from the feeder on the stem of the T, referred to as the “base feeder,” the rat made a choice to enter one of the two reward arms of the T. After one reward arm was chosen (i.e., rats approach within roughly 15 cm of the reward site), an automated gate on the opposite choice arm would raise, blocking the rat from choosing the other arm. The gate on the return arm was also lowered at this time to allow the rat to return to the base feeder and hence start the next trial. Once a rat was at the base feeder, the return gate raised, preventing backwards navigation.

#### Weight-lifting

A novel weight-lifting effort-based decision-making task was conducted on a Y-shaped maze, painted in light gray epoxy paint, with 51 cm high black corrugated plastic walls. The base arm of the maze was a total of 76 cm long while the two choice arms were 37 cm long. The running track was 15 cm wide. In each choice arm, a seesaw lever (30.5 cm long × 2.54 cm wide × 0.5 cm thick) protruded through the back wall (Figure 2C). Small copper pellets were packaged into plastic bags and attached to the back end of one lever to control the force required to depress the lever. Position was monitored via a magnet and Hall effect sensors. Food delivery wells were located in front of each lever and near the end of the base arm. A small LED cue light, positioned 24 cm from the floor, illuminated when a successful lever press was made. In the base arm, an infrared emitter-sensor pair were used to detect the rat's presence and deliver food when appropriate. The experiment was conducted in a dimly lit room (as above) using an infrared-sensitive video camera and infrared lights for behavioral monitoring.

The stem of the Y was referred to as the “base” and rats were trained to alternate between the base feeder, where a photo beam interrupt delivered food, and one of the two choice arms, where a lever press was required to yield a reward.

#### Courage task

A newly developed courage task was also conducted on a Y-maze, painted in light gray epoxy paint, with 35.6 cm high black corrugated plastic walls. All three arms of the maze were 76 cm long and 14 cm wide. The maze was elevated 60 cm from the ground. Infrared beams were used on all three arms to trigger reward delivery and track trials. On one of the reward arms, the wood floorboard and walls could be removed, revealing a wire mesh bridge, exposed on the sides and open to the bottom (Figure 2D). In Experiment 1, this task was conducted in a dimly lit room (as above). During Experiment 2, this task was conducted under standard room lighting, provided by four 40 W fluorescent tubes.

As in the weight-lifting task, the stem of the Y was referred to as the “base” and rats were trained to alternate between the base feeder and one of the two choice arms. On each arm, a photo beam across the path 10 cm from the feeder well was used to detect the rat's presence and trigger reward delivery.

#### Open field test

Rats in Experiments 1 and 2 were tested on the open field test after completion of all experimental tasks, conducted on a 123 × 123 cm square field made of corrugated plastic with 51 cm high walls. Rats were placed on the open field and allowed to explore for 5 min. The task was videotaped and an in-house program allowed for analysis of distance covered, time spent in periphery vs. center, and running speed.

### Training and testing procedures

#### Ramp-climbing task

In Experiment 1, rats were trained on the ramp climbing task in the morning and the lever pressing experiment in the afternoon. Pre-training on both tasks continued until rats had reach behavioral criterion or until 28 days had elapsed, whichever came first. The stages of training are described below.

Rats were first pre-trained to run for reward on the maze with both ramps at zero height. In this stage, all pulse widths (PW; the time the solenoid valve was open to let fluid run through) were set at equal durations (600 ms), which produced a medium amount of food reward. Once rats reached >30 trials on the task, the base feeder PW was lowered to 300 ms, which produced a small amount of food reward. After rats successfully completed 60 trials in one session, they moved on to reward contingency learning. During pre-training and all subsequent testing, each session lasted 20 min or 60 trials, whichever came first.

In the next stage rats learned to choose a high reward over a low reward. For each rat, a HRA and a LRA were assigned and stayed fixed throughout the remainder of the experiment. Left and right arms were assigned as HRA in a counter-balanced fashion, subject to the constraint that rats with a strong side bias were assigned a HRA on the non-preferred side. Based on the solenoid PW, the HRA:LRA reward ratio was 4:1. That is, the LRA PW was 300 ms and the HRA PW 1200 ms. Due to the non-linear response of delivery system, however, the volume and calorie ratio was approximately 12:1. The volume at 300 ms was 0.02 mL (0.02 kcal), at 600 ms it was 0.1 mL (0.11 kcal), and at 1200 ms it was 0.24 mL (0.26 kcal). Rats had to reach a criterion of 80% or greater HRA trials for 2 non-consecutive days before moving on to effort training.

In the final pre-training stage, rats were gradually acclimated to increasing ramp height on the HRA. For all stages in effort training and subsequent testing, rats received four forced trials without any effort present, two to each arm in alternation, at the beginning of each session. These trials were not counted in any analysis and simply served to remind the rat of the reward contingencies. This is consistent with methodology used in previous studies (Walton et al., 2003). After the four forced trials, the rat was taken off the maze while the ramp of the HRA was raised to the specific height for the session. In effort stage 1, rats were trained to climb a 15.2 cm high ramp, followed by a 25.4 cm high ramp in stage 2, 30.5 cm in stage 3, and 35.6 cm in the final effort stage 4. In stages 1–3, rats had to achieve a criterion of 1 day of 60 trials and 80% of HRA entries or greater. During this training phase, rats who failed to achieve criterion within 5 days at any given stage were moved to the next stage. In stage 4, rats had to achieve a criterion of 2 days of criterion performance, not necessarily consecutive.

Following surgery, rats were allowed to recover for 10–14 days before testing and then received 3 days of testing on the ramp-climbing task. Here, rats received four forced trials without effort as previously described. Then the ramp height of the HRA was raised to 35.6 cm. During testing, rats again completed the ramp-climbing session in the morning and the weight-lifting sessions in the afternoons.

As described in the Experimental Overview Section, two additional ramp tasks followed ramp-climbing testing. On the incremental task, rats received a number of height increments in the HRA within one single session, where heights were incremented every 10 trials. Increments consisted of 0, 15.2, 25.4, 30.5, 35.6, 40.6, 45.7, and 50.8 cm. On the challenge task, rats first received a zero effort control day to re-establish their reward learning. In the second session on the following day, rats received the four forced trials at 0 cm, followed by the remainder of the session at 50.8 cm, the highest height they had previously experienced.

#### Weight-lifting lever task

In Experiment 1, rats were pre-trained on the ramp and lever tasks simultaneously, as described above. In Experiment 2, rats were pre-trained on levers only. In both Experiment 1 and 2, training proceeded until criterion was achieved (as described below) up to a maximum of 28 sessions. In Experiment 3, rats were pre-trained on the lever task with unweighted levers until criterion up to a maximum of 14 days.

Rats were first pre-trained to enter arms and depress levers for reward with no weights on the levers. At this stage, the training protocol, including amounts of food and performance criteria were exactly as described for the ramp pre-training except that the base reward port was now on the stem of the Y. To help shape lever pressing behavior, during the initial period with 600 ms PW at all reward sites, a slight press of the lever was sufficient to trigger reward. After this period, rats had to depress the lever through 60 degrees of arc (roughly 14 cm) to receive reward. As with the ramp task, each pre-training and testing session lasted 20 min or 60 trials, whichever came first.

After rats reached 60 trials in 20 min, they moved on to reward contingency learning and HRA/LRA were determined the same way as described above for the ramp-climbing task. After the four forced trials, the rat was blocked from access to the decision arms while a weight was affixed to the end of the seesaw lever. Weights were tailored to each individual rat's target weight, expressed as a percent of body weight and rounded up or down to the nearest 5 g. In effort stage 1, rats were trained on a weight of 10% of their body weight. Once a minimum of 80% HRA trials was reached in a session, rats moved on to the next stage with the same criterion. In Experiment 1, throughout stages 2–4, rats were trained on 15, 17.5, and 20% of their body weight, respectively. In Experiment 2, throughout stages 2–8, rats were trained rats on 15, 20, 25, 30, 32.5, 35, 37.5, and 40% of their body weight. In the final stage, rats had to achieve two sessions of criterion performance, though not necessarily on consecutive days. During testing after surgery, rats were first presented with the four forced trials, and then directly with a weight of 20% (Experiment 1) or 40% (Experiment 2) of their body weight on the HRL.

In all three experiments, rats were allowed to recover for 10–14 days after surgery before testing. In Experiment 1 and 2, rats received 2–3 lever-pressing testing days followed by an equate effort day, and an incremental session. In the incremental task, rats received a number of weight increments in the HRA within one single session, where weights were incremented every 10 trials. Increments consisted of 0, 15, 20, 25, 27.5, 30, 32.5, 35, 40, 45, and 50% of the rats' body weights, depending on the experiment. In Experiment 1, rats also underwent a lever challenge task following courage testing. Rats first received a zero effort baseline day to re-establish their reward learning. In the second session on the following day, rats received the four forced trials at 0%, followed by the remainder of the session at 40%, the highest weight they had previously experienced. In Experiment 3, rats were only pre-trained on lever pressing and reward contingency with no weights attached. After surgery, they were then tested on the incremental effort task.

#### Courage task

Pre-training and testing for this task were conducted entirely post-surgery. Rats completed pre-training and reward contingency training, as in the previous two tasks, with walls on all three arms. On this task, rats were started with a base PW of 300 ms with the LRA delivering 300 ms worth of Ensure and the HRA delivering 1200 ms worth of Ensure. Once rats achieved two sessions of 60 trials and 80% HRA, walls and floorboard of the HRA were removed creating an exposed, and presumably scary, bridge to the high reward well. In Experiment 2, the room lights were also turned on that this point.

### Surgery

All rats underwent surgery. In Experiment 1, rats were matched according to task performance on the ramp-climbing task, factoring in both days to criterion and total HRA choices on the final day, and then divided into sham and ACC lesion groups prior to surgery. In Experiments 2, rats were matched according to task performance on the weight-lifting task. In Experiment 3, matching was based on task performance on the reward-discrimination training.

Thirty minutes prior to commencing anaesthesia, rats were injected with 0.03mg/kg buprenorphine (concentration: 0.03 mg/mL, Sigma Aldrich, Oakville, ON) to increase their pain threshold. Isoflurane (Abbott Laboratories, Abbott Park, IL) was set between 1 and 3% throughout the duration of the surgery so as to maintain a constant respiration rate. All rats received a 3 mm wide by 5 mm long craniotomy, exposing the dura. For sham rats, the craniotomy was then covered with a thin film of brain butter (one part bone wax and two parts mineral oil). For lesion rats, bilateral injectors, 28 gauge with a 45 degree bevel, were lowered to each of the following coordinates (after Walton et al., 2002): (anterio-posterior/dorso-ventral/medio-lateral): +3.0/−1.5/±0.75 mm, +2.3/−2.0/±0.75 mm, +1.6/−2.0/±0.75 mm, +0.9/−2.0/±0.75 mm, +0.2/−2.0/±0.75 mm. Anterior-posterior is measured from bregma and depths are relative to dura surface. Injectors were connected via tubing (thin-walled PE50 tubing, Fisher Scientific, Toronto, ON) to two Hamilton syringes (Hamilton, Reno, NV) held in a syringe pump (Legato 100 Series, KD Scientific, Holliston, MA). Injections of N-Methyl-D-aspartic acid (NMDA, 300 μL, 15 mg/mL, Sigma Aldrich, Oakville, ON) were made at a rate of 0.15 μL/min and injectors were left in place for 5 min after injection to allow for diffusion. Following the final injection, the craniotomy was covered with a thin film of brain butter prior to suturing. Post-surgery, rats were treated with 1 mg/kg Metacam (meloxicam, concentration: 5 mg/mL, Boehringer Ingelheim, ON) for 3 days at 24 h intervals.

### Histology

Following completion of all experiments, rats were deeply anaesthetized with 100 mg/kg sodium pentobarbital, then transcardially perfused with 1 × phosphate-buffered saline (PBS) and 4% paraformaldehyde (PFA). Brains were post-fixated in 4% PFA for at least 48 h before they were transferred to a 30% sucrose solution with sodium azide. Brains were sectioned at 40 μm on a cryostat and stained with 0.5% Cresyl violet. Sections were imaged using a nanozoomer (Hamamatsu Corporation, Middlesex, NJ).

### Analyses

For any statistical test, if the sphericity assumption was violated as judged by Mauchly's test of sphericity, the Greenhouse–Geisser ε values were considered. For any value below 0.7, the values from the Wilks' λ row in the multivariate table are reported. All tests were conducted at an alpha level of 0.05. In any ANOVA, planned pairwise comparisons were used with the Bonferroni adjustment for comparison of main effects. For multiple *post-hoc* comparisons, the Bonferroni correction was used to adjust the alpha level.

## Results

### Histology

**Figure 3:**
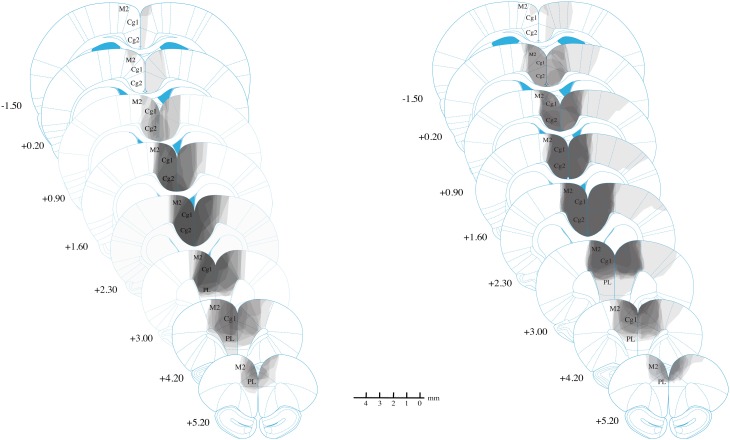
**Reconstruction of anterior cingulate lesions**. Lightest gray indicates damage in only one animal while darkest gray indicates damage common to all animals. Experiment 3 results are not shown but are highly similar to those shown for Experiment 2. Lesions are largely restricted to Cg1 and Cg2, with minor damage to M2 and PL.

The majority of lesions in all experimental were fairly restricted to Cg1/Cg2, with almost all cases including minor damage to M2 and PL (see Figure 3). Lesions could extend as far anterior as +5.2 mm AP from bregma, damaging parts of PL/IL and M2 anterior to the Cg1. Corpus callosum was generally intact. No lesions extended posteriorly past −1.56 mm AP from bregma, and many did not extend this far. Overall, there an anterior shift of lesions relative to our injection sites, suggesting a slight mismatch between our Long-Evans rats and our reference atlas(Paxinos and Watson, 2007). Consequently, in the atlas coordinates, damage tended to extend anteriorly, but was minimal posteriorly, even at the exact location of the posterior injection (+0.24 mm).

In the lateral direction, damage rarely extended into M1, although in one animal with extensive damage, the lesion reached into primary somatosensory cortex on one side. This animal, however, was not excluded because it showed normal behavior on the open field test (data not reported).

### Ramp-climbing task

**Figure 4:**
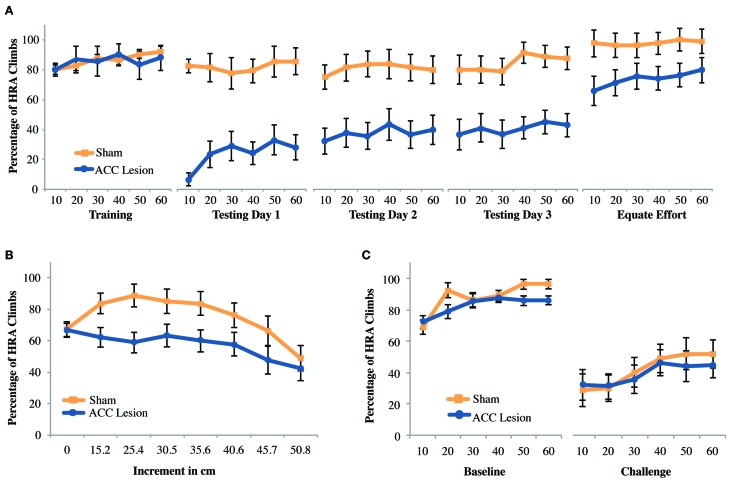
**Mean performance of ACC lesioned and sham control rats on all ramp-climbing effort tasks**. **(A)** Percentage of high-reward arm (HRA) successful climbs in which a 35.6 cm ramp was present on the HRA across 5 testing sessions. Means and standard errors are first computed in blocks of 10 trials for each animal and then averaged within groups. “Training” shows performance on the last day of training before surgery. Testing days 1–3 show performance post-surgery. “Equate Effort” shows performance when a 35.6 cm ramp was present in both high- and low-reward choice arms. **(B)** Incremental test results showing the percentage of HRA climbs as ramp height was increased from 0 to 50.8 cm within a single session. **(C)** Challenge test results showing the percentage of HRA climbs when ramp height on the HRA was suddenly increased from 0 during the baseline session to 50.8 during the subsequent challenge session.

We aimed to confirm that ACC lesions deter high-reward choices when that reward requires climbing a ramp, as has previously shown (Walton et al., 2002). After exclusions due to health-related issues and lesion size a total of 19 animals were included in the experiment. Of these, another two were excluded due to inadequate performance during pre-training. Hence, for this analysis data from 9 rats with ACC lesions and 8 rats with sham surgery was compared. Results are shown in Figure 4A. To facilitate analysis, data from each testing session was analyzed by trial within a session, using averages over blocks of 10 trials. We compared performance between groups on the last day of training and first day of testing using a repeated measures analysis of variance (ANOVA) with trial (trial bins 10, 20, 30, 40, 50, 60) and session (last day of training, testing day 1) as the within-subjects factors and Group (ACC lesion, sham) as the between-subjects factor. The analysis revealed a significant Session × Group interaction, *F*_(1, 15)_ = 22.86, *p* < 0.001 *Post-hoc* tests for simple main effects showed that ACC-lesioned rats performed significantly more HRA climbs on the last day of training (*M* = 85.56, *SD* = 5.34) than on the first day of testing (*M* = 24, *SD* = 29.82), *F*_(1, 8)_ = 32.4, *p* < 0.001. Most importantly, ACC-lesioned rats performed significantly fewer HRA climbs on the first testing day (*M* = 24, *SD* = 29.82) than sham animals (*M* = 81.88, *SD* = 9.23), *t*_(9.69)_ = 5.53, *p* < 0.001. These results replicate existing results on standard ramp-climbing paradigms after ACC lesions (e.g., Walton et al., 2002, 2003).

As can be seen in Figure 4A, results suggested that lesion animals gradually increased their HRA climbs across days, as has been observed in other studies (Walton et al., 2002). However, a repeated measures ANOVA of trial bins across the 3 testing days (a total of 18 trial bins) with Group as a within subjects factor showed main effects of both trial and group but no Trial × Group interaction [Trial: *F*_(4.28, 64.26)_ = 2.89, *p* = 0.026; Group: *F*_(1, 15)_ = 20.48, *p* < 0.001]. Hence, although both groups, combined, improved over time there was no statistical evidence that lesioned animals, in particular, improved with time.

As with previous ramp-climbing studies, a final session assessed rats' ability to make decisions about high and low reward when both arms had a high (35.6 cm) barrier (the “equate effort” test). If lesioned rats were to successfully complete this task, it would confirm that (1) they can make correct decisions about reward magnitude and (2) they are capable of climbing high ramps, helping to rule out gross motor deficits as an explanation for their avoidance of high ramps in the high-ramp/no-ramp test. Compared to the last day of training, equating effort caused both groups to choose the HRA more frequently. However, it did not eliminate the differences between groups. These conclusions are borne out by a Session × Trial × Group ANOVA which showed main effects of both session and group [Session: *F*_(1, 15)_ = 21.84, *p* < 0.001; Group: *F*_(1, 15)_ = 11.64, *p* = 0.004] but no Session × Group interaction. This result suggests that rats with ACC lesions have decision-making deficits beyond those involved in weighing effort and reward. The most likely explanation is that, owing to the extensive number of testing trials during which lesioned rats choose the LRA, lesioned rats were unable to break habitual patterns of response.

To assess the effects of different ramp heights on the decision making abilities of rats with ACC lesions and sham controls, all rats were tested in an incremental session, where the effort was incremented every 10 trials. A repeated measures ANOVA with the within-subjects factor increment (8 increments) and the between-subjects factor group revealed a main effect of increment, *F*_(7, 9)_ = 4.23, *p* = 0.024, but none of group, and no significant interaction. Pairwise comparisons showed that overall performance across groups at 0 cm was significantly worse than at 15.2, 25.4, and 30.5 cm, but performance on these latter three increments as well as 35.6 and 40.6 cm was significantly better than on 50.8 cm, all *p*-values <0.028 (Figure 4B). Hence, combined across groups, rats showed gradually increasing HRA climbs during the first half of the session, probably due to practice effects, followed by a gradual decline in HRA climbs as effort levels increased. A follow-up ANOVA excluding the 0 cm condition showed a marginally significant effect of group [*F*_(1, 15)_ = 4.54, *p* = 0.05], but again no group × increment interaction. Hence, while there is evidence that lesioned animals performed worse on this task they do not reduce HRA choices any faster as effort increases.

Based on evidence that medial prefrontal regions are critical to adjusting to changing task contingencies (McDonald et al., 2008), we wondered whether the large differences in performance on the first testing day after surgery (Testing Day 1) could be attributed to the sudden presentation of a large ramp after more than a week without practice on the task. To examine this possibility, rats were retrained to discriminate high and low rewards without barriers and then, on a subsequent testing day, were suddenly presented with a very high (50.8 cm) ramp in the HRA. As shown in Figure 4C, this manipulation caused a strong reduction in HRA climbs in both groups, but no differences between groups. A Session × Trial × Group ANOVA showed a significant main effect of Session [*F*_(1, 15)_ = 63.76, *p* < 0.001] but no other significant differences. Hence, we found no evidence that a sudden, unexpected increase in ramp height leads to specific behavioral impairments in rats with ACC lesions.

**Figure 5:**
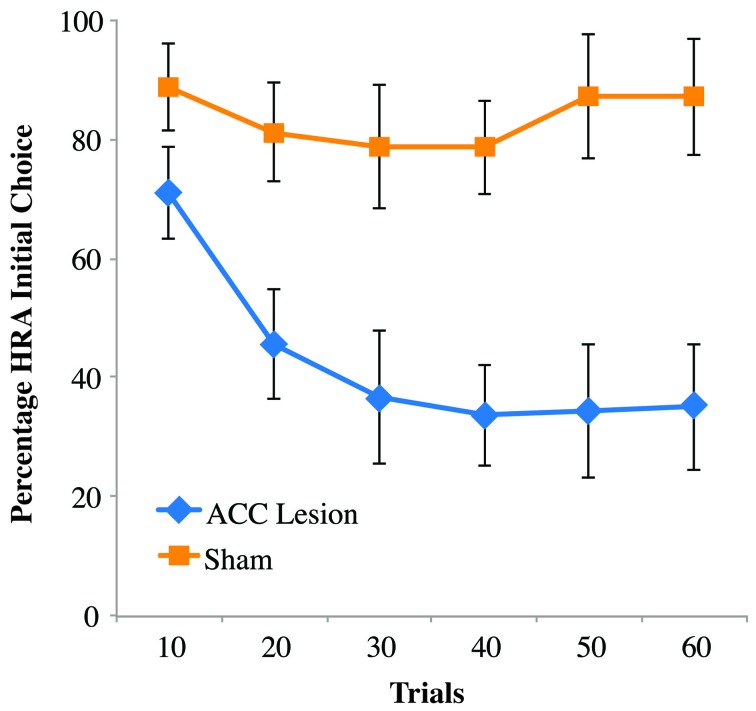
**Percentage of high-reward arm turns, independent of climb on the first post-surgical testing session of the ramp task**. Shown are the percentage of times rats turned into the HRA, whether or not that turn was followed by a successful climb. Data are presented in blocks of 10 trials. Error bars are standard error of the mean. In the initial block of 10 trials, rats with ACC lesions were almost as likely to turn into the HRA as sham controls, but far less likely to actually climb the ramp once they encountered it.

Looking more closely at rats' behavior on the first day of testing, it was noticed that ACC-lesioned rats behave differently than sham animals. Specifically, lesion rats were observed turning into the HRA without climbing the ramp and instead turning around and ultimately choosing the LRA. In many instances, the lesioned rats actually put their paws upon the ramp as if to start climbing, but then backed away. To quantify this behavior, previously acquired video of rat's performance on Testing Day 1 was scored for the frequency of initial turns into the HRA whether or not that turn ultimately resulted in a successful climb. The results are shown in Figure 5. Comparing these results to those shown in Figure 4A reveals a striking difference. In the first 10 trials, lesioned rats turned into the HRA 71 percent of the time but only climbed the ramp 7 percent of the time. Sham controls, in comparison, chose the HRA 89 percent of the time and climbed the ramp 83 percent of the time. A Trial Bin × Group ANOVA showed a significant main effect of Trial [*F*_(5, 55)_ = 7.810, *p* < 0.001] and a Trial × Group interaction [*F*_(5, 55)_ = 5.129, *p* < 0.005]. *Post-hoc* paired comparisons of groups within trials showed a significant difference for trial bins 20, 30, 40, and 50 (using a Bonferoni corrected alpha of 0.008) but not for bins 10 (*p* = 0.052) and 60 (*p* = 0.015). Even without correcting for multiple comparisons, the results show no statistical difference between HRA turns in the first 10 trials, suggesting that rats with ACC lesions do not have an impairment in choosing the HRA but rather in following through on the climb once that arm is chosen. Interestingly, however, after repeated trials they do stop turning into the HRA and instead turn first into the LRA.

### Weight-lifting task

#### Experiment 1: weight-lifting with moderate weight

**Figure 6:**
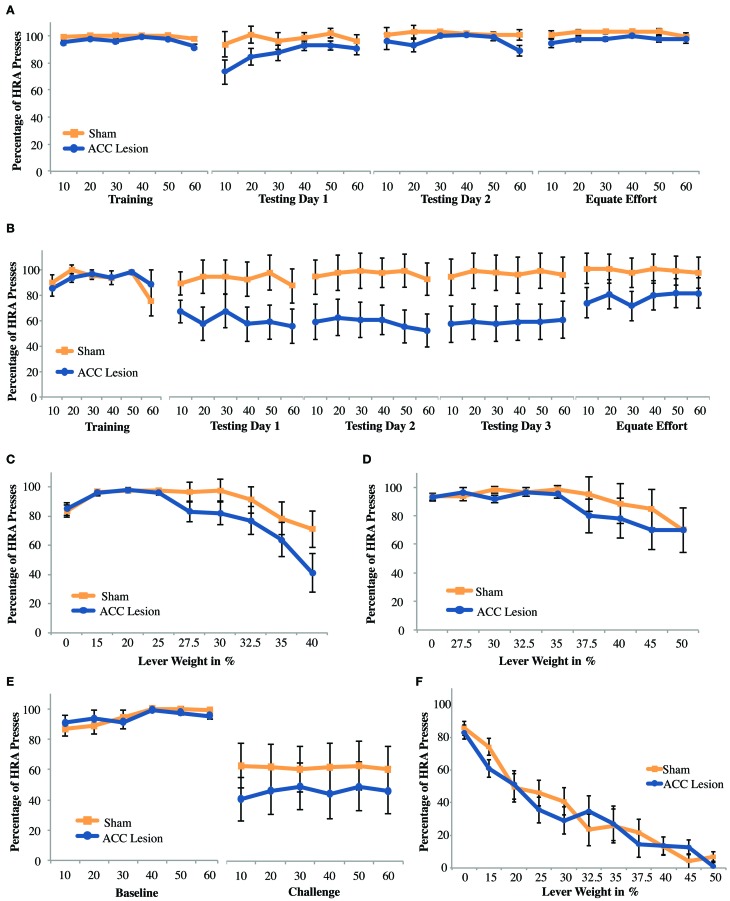
**Mean performance of ACC lesioned and sham control rats on all weight-lifting effort tasks**. **(A)** Percentage of high-reward arm (HRA) lever presses for 4 session in which a weight of 20% of body weight was present on the high-reward lever. Means and standard errors are first computed in blocks of 10 trials for each animal and then averaged within groups. “Training” shows performance on the last day of training before surgery. Testing days 1–2 show performance post-surgery. “Equate Effort” shows performance when both levers were weighted with 20% of body weight. **(B)** Percentage of HRA lever presses for sessions in which a weight of 40% of body weight was present on the high-reward lever. These results are from Experiment 2. **(C,D)** Incremental test results showing the percentage of HRA lever presses as lever weight was incrementally increased within a single session. Results in **(C)** are from Experiment 1 and **(D)** from Experiment 2. **(E)** Challenge test results from Experiment 1 showing the percentage of HRA lever presses when lever weight on the HRA was suddenly increased from 0 during the baseline session to 50% during the subsequent challenge session. **(F)** Incremental test results for rats with no prior weight-lifting experience. These results are from the cohort of animals tested in Experiment 3 in which rats were pre-trained to press the high-reward lever without weights before surgery and tested with incrementally increasing weights in a single session after surgery.

The purpose of this experiment was to test whether rats with ACC lesions would show impairments in effort-reward decisions when effort involved pressing a weighted lever. The experiment was conducted in parallel with the ramp task, using the same group of animals, a total of 10 rats with ACC lesions and 9 sham operated controls. We predicted that we would see a similar effect on this task as on the ramp-climbing task, namely, ACC-lesioned rats would greatly reduce their choices of the HRA after surgery. However, as can be seen in Figure 6A, that was not the case. A repeated measures ANOVA with the within-subjects factors Trial (trial bins 10, 20, 30, 40, 50, 60) and Session (last day of training, testing day 1) and the between-subjects factor Group revealed a significant Trial × Session × Group interaction, *F*_(5, 13)_ = 3.81, *p* = 0.024. *Post-hoc* tests showed a significant group difference on the last day of training, where ACC-lesioned rats (*M* = 96.1, *SD* = 3.03) performed worse than sham animals (*M* = 99.33, *SD* = 1), *t*_(11.12)_ = 3.18, *p* = 0.009. The apparent group differences on testing day 1 proved non-significant. These results show that the two groups were not equivalent before surgery, a result which is not unexpected as rats were assigned to groups based on their performance on the ramp task, not the lever task. More importantly, there was no evidence that lesioned rats were less likely to choose the HRA when tested after surgery.

To compare group performance across testing days, a repeated measures ANOVA with the within-subjects factor Trial (12 levels for each of 6 trial bins over 2 testing days) and the between-subjects factor Group was conducted. This analysis revealed no significant differences, indicating that groups did not differ from each other on either testing day and that performance across testing days was stable. When comparing testing day 2 to the equate effort day in a repeated measures ANOVA as outlined above, a small but significant main effect of group was found, *F*_(1, 17)_ = 5.29, *p* = 0.034, indicating that sham animals (*M* = 98.8, *SD* = 1.26) consistently outperformed ACC-lesioned animals (*M* = 94.09, *SD* = 3.32). Given the small size of the effect, these results may simply reflect the pre-existing differences in performance seen during training before surgery. More importantly, there was no Session × Group interaction.

The very high percentage of HRA choices by both groups observed in the 2 testing days raised the possibility that the weight used in the first task was simply not heavy enough. To test whether higher levels of effort might cause lesioned animals to stop choosing the HRA, rats were further tested in an incremental task in which the weight attached to the lever was increased every 10 trials. As can be seen in Figure 6C, increasing the weight caused both groups of animals to reduce choices of the HRA. At higher weights, lesion animals chose the HRA slightly less than sham controls. However, an Increment × Group ANOVA showed only a main effect of increment, *F*_(8, 10)_ = 3.48, *p* = 0.034. The main effect of Group and the Increment × Group interaction were both non-significant. Pairwise comparisons showed that, overall, rats performed significantly fewer HRA entries at increment 0% compared to 15, 20, and 25%, but performed significantly more HRA entries at those latter three increments compared to the 40% increment. As with the ramp results, these findings suggest that both groups increased their HRA responses during the first dozen trials but then reduced their HRA responses as lever weight increased.

Finally, parallel to the ramp-climbing study, rats were tested with a sudden, unexpected high level of effort in the “challenge task.” Rats were re-trained with unweighted levers during one session, allowing both groups to re-learn the HRA, and were tested in the next session with a weight that was 40% of their body weight. This was the highest weight they had previously experienced. As with previous manipulations, there was a slight tendency for ACC lesion animals to choose the high-effort/high-reward lever less than sham controls (Figure 6E). However, a Session × Trial × Group ANOVA showed a main effect of Session *F*_(1, 17)_ = 18.86, *p* < 0.001, but no effect of Group or Session × Group interaction.

#### Experiment 2: weight-lifting with heavy weight

In the first weight-lifting test in Experiment 1 (Figure 6A), both groups choose the HRA at very high rates, suggesting a ceiling effect due to insufficient weight. Thus, the task was repeated with a new group of animals using a higher effort level (i.e., 40% of body weight). After exclusions due to health-related issues and lesion size a total of 20 animals were initially included in the experiment. The weight-lift task proved to be extremely difficult and only 12 animals successfully completed pre-training. Hence, testing was limited to 6 rats with ACC lesions and 6 rats with sham lesions. As can be seen in Figure 6B, larger effort did reduced the degree to which ACC lesioned animals chose the HRA during testing compared to Experiment 1. However, a Trial × Session × Group ANOVA comparing the last day of training to the first day of testing showed no significant main effects or interactions. Similarly, a Trial × Group ANOVA across the 3 testing days yielded no significant main effects or interactions. Finally, a repeated measures ANOVA comparing testing day 3 and the equate effort session following the design previously used also showed no significant main effects or interactions. Hence, despite the apparent differences in group means, all statistical tests indicate that lesion animals perform no differently than sham controls.

**Figure 7:**
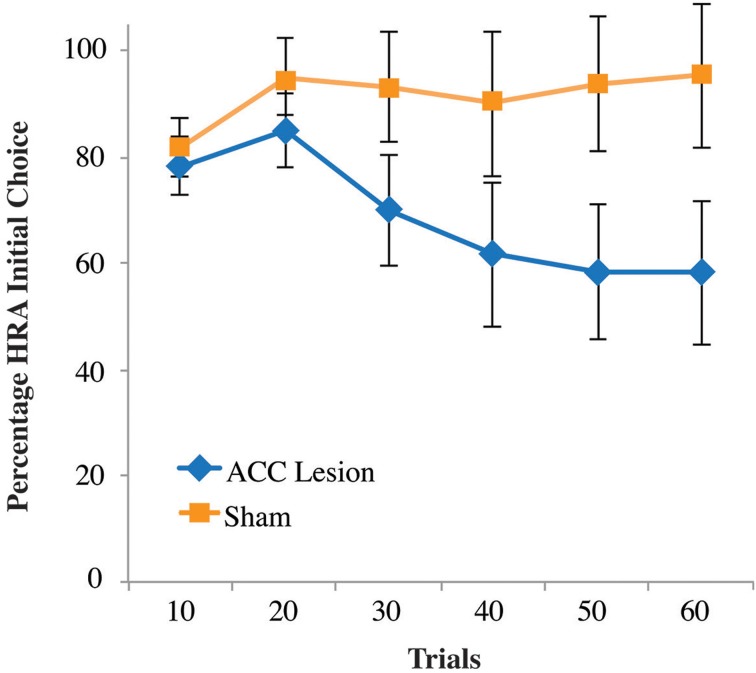
**Percentage of high-reward arm lever choices, independent of successful lever press on the first post-surgical testing session of the high-weight lever test, Experiment 2**. Shown are the percentage of times rats turned into the HRA and placed their paws on the lever, whether or not that action was followed by a successful lever press. Data are presented in blocks of 10 trials. Error bars are standard error of the mean.

Closer examination of our results revealed why no statistical differences were observed between groups. As shown in the supplementary material, Figure S2, 4 lesion rats pressed the high-reward lever the majority of the time, similar to our sham control rats. The remaining two lesioned rats pressed the HRA a few times and then their HRA responses dropped to near zero. Hence, the data show a binary effect with some rats looking no different than controls and two rats showing a dramatic drop-off in HRA lever presses. Re-examination of video data showed that these two rats usually chose the HRA during the first 10–20 trials but were usually unable to depress the lever to its lowest position and hence trigger reward delivery. After many failed attempts, these two rats switched away from the HRA and seldom tried again. This pattern is reflected in the choice data shown in Figure 7. This graph shows the number of times rats entered the HRA and put their paws on the lever, whether they were successful in pressing the lever all the way down or not. During the first 10 trials, it is apparent that both lesion and control animals initially chose the HRA lever. However, after 20 trials, the two lesion animals who could not succeed on the lever press switched away from choosing the HRA, leading to a reduced mean HRA choice. However, a Trial × Group ANOVA showed no significant differences.

The same group of animals was also tested in an incremental session, this time using weights up to 50% of body weight (Figure 6D). Despite an apparent trend for both groups to respond less to the HRA with increasing weight, no significant main effects or interactions were found. Importantly, all of the weight-lifting tests in Experiment 2 were done with a relatively small number of animals and hence our power is low.

#### Experiment 3: weight-lifting without prior training

The results of Experiment 2 showed that our method of measuring effort-reward decision making with weighted levers had significant drawbacks. Specifically, increasing the weight to a level which might discourage HRA choices meant that few animals could successfully achieve our pre-training criterion level of 80% HRA lever presses. A partially depressed lever also fails to yield reward, leading two rats to apparently assume that the HRA was non-functional and leading them to choose the LRA instead. This may account for the binary outcomes observed within our lesion group. To overcome these problems, a third experiment was conducted using an incremental weighted lever test. Importantly, this incremental test was the first test conducted after surgery, reducing the possibility that previous experience could have led to compensatory adjustments in lesioned animals.

Another consideration in the design of this experiment was our observation that previous weight training shifted the break-point of lever effort-discounting curves. In Experiment 1, rats were pre-trained to lift up to 20% of their body weight. When these rats were tested with incrementally increasing values, HRA choices began to drop when weights reached 30%. In contrast, when rats were pre-trained with up to 40% of their body weight in Experiment 2, there was no statistically significant fall-off in responses, even at 50% of body weight. Hence, we hypothesized that testing rats in an incremental test with no prior experience lifting weights might lead to steeper effort discounting and hence be the most sensitive measure of whether ACC lesioned animals were as willing as control animals to exert effort to achieve reward.

Twenty four animals were included in the study, 12 rats with ACC lesions and 12 rats with sham surgery. As shown in Figure 6, both groups of animals showed a much steeper drop-off in HRA choices as weight increased than was observed in either Experiment 1 (Figure 6C) or Experiment 2 (Figure 6D). More importantly, there were no differences between groups. These observations are borne out by statistical analyses. An Increment × Group ANOVA showed only a main effect of increment, *F*_(10, 13)_ = 57.68, *p* < 0.001 but no effect of Group or Increment × Group interaction. Hence, when tested without prior weight-lifting experience, increasing weights clearly deterred HRA choices. However, despite the increasing difficulty of the task, we found no indication that rats with ACC lesions were less likely to choose the high-effort/high-reward lever. We conclude that prior training cannot explain the lack of group differences in our previous lever-pressing tests.

### Courage task and open-field behavior

#### Experiment 1: courage with moderate fear

**Figure 8:**
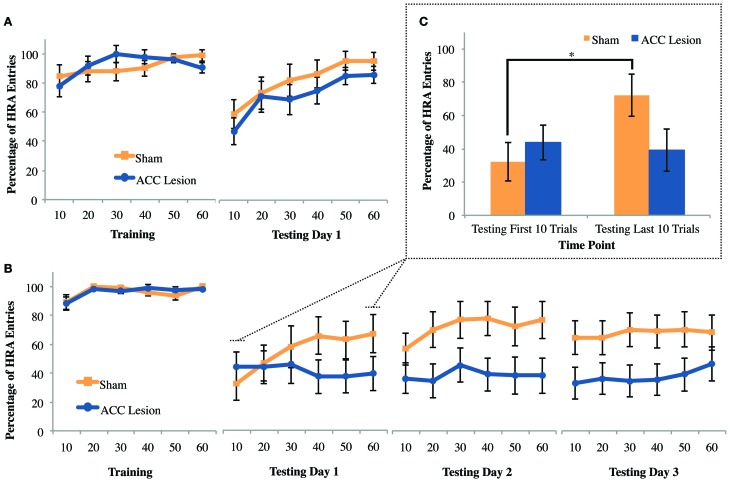
**Mean performance of ACC-lesioned and sham control rats on all courage tasks. (A)** Percentage of high-reward arm (HRA) choices for the last day of training and first day of testing on the courage task. Rats were trained with walls and a solid floor and then tested with those walls and floor removed. These results are from Experiment 1, which was conducted in a dimly lit room. Means and standard errors are first computed in blocks of 10 trials for each animal and then averaged within groups. **(B)** Percentage of HRA choices for the last day of training and 3 days of testing on the courage task in Experiment 2. For all testing days, the walls and floorboard were removed from the HRA and the room lights turned on. **(C)** Comparison of mean HRA choices during 10 trials and last 10 trials on Testing Day 1 in Experiment 2. Asterisk indicates a significant difference between first and last 10 trials for sham controls.

The question that motivated the courage task was whether other forms of cost, such as the need to overcome fear, could deter HRA choices in ACC lesioned animals in the same way that physical effort deterred HRA choices on the ramp-climbing task. To answer this question, rats were trained to choose between two arms of a maze that differed in reward. Then, during testing, the walls and floorboards of the HRA were removed, creating an exposed bridge that rats instinctively avoid. If the ACC mediates all forms of cost-benefit decisions, then rats with ACC lesions should be more deterred by the high-fear/HRA than sham controls. This experiment was conducted after the Experiment 1 ramp and weight-lifting tasks using the same group of animals (10 rats with ACC lesions and 9 rats with sham surgery). As shown in Figure 8A, exposing the HRA caused a definite reduction in HRA choices in both groups. Further, both groups increased their HRA choices across the testing session, suggesting habituation to the fear-inducing arm. However, there were no apparent differences between groups during testing. These effects were supported by statistical tests. A Trial × Session × Group ANOVA comparing the last training day to the testing day revealed significant main effects of Trial, *F*_(5, 13)_ = 8.96, *p* = 0.001, and Session, *F*_(5, 13)_ = 11.06, *p* = 0.004, but no main effect or interactions involving Group. Pairwise comparisons showed that across groups and sessions, rats performed significantly worse in trial bin 10 compared to all other trial bins, all *p*-values <0.008. Further, all rats performed significantly more HRA entries during the last day of training (*M* = 91.77, *SD* = 6.84) compared to testing day 1 (*M* = 77.11, *SD* = 2.72). These results show that exposing the HRA was sufficient to deter HRA choices but did not cause any behavioral differences between groups.

Behavioral observations indicated that rats often entered the HRA after choosing the LRA before returning to the base zone on the stem of the Y. However, quantitative analyses showed that this behavior was no more likely in control animals than lesion animals. Hence, it merely indicates that the task was not as fear-inducing as results based on initial choice, reported above, suggest.

#### Experiment 2: courage with more intense fear

The courage task in Experiment 1 showed no behavioral difference between rats with ACC lesions and sham controls. However, the reductions in HRA entries upon exposure of the HRA were rather modest, raising the possibility that greater levels of fear might selectively deter rats with ACC lesions from the HRA. Thus, the level of anxiety (and hence required level of courage) was increased by turning on the room lights, a manipulation known to increase the anxiety of rodents in the elevated plus maze (Hogg, 1996). This test was conducted using the same group of animals previously tested in the weight-lifting task in Experiment 2, consisting of 20 animals, 11 with ACC lesions and 9 sham controls. As shown in Figure 8B, turning on the room lights caused both groups to avoid the high-fear/HRA to a greater degree than in Experiment 1. A Trial × Session × Group ANOVA comparing the last training day to the first testing day revealed a significant Trial × Session × Group interaction, *F*_(5, 14)_ = 5.12, *p* = 0.007. *Post-hoc* analyses revealed a simple main effect of trial within the control group, *F*_(5, 40)_ = 5.93, *p* < 0.001, but pairwise comparisons were non-significant. Further, *post-hoc* tests indicated a simple main effect of session within the control group, *F*_(1, 8)_ = 12.06, *p* = 0.008, where sham rats performed significantly more HRA entries during the last day of training (*M* = 96.11, *SD* = 4.43) than during testing 1 (*M* = 55.28, *SD* = 13.46). The same was true for ACC-lesioned rats, *F*_(1, 10)_ = 24.27, *p* = 0.001; animals performed significantly more HRA entries during the last day of training (*M* = 96.21, *SD* = 4.04) than during testing 1 (*M* = 41.06, *SD* = 3.61). No other *post-hoc* tests were statistically significant. Hence, the level of exposure in the HRA was clearly sufficient to deter choices but both lesion and control animals were equally deterred. To compare group performance across the 3 testing days, a Trial × Group ANOVA was conducted. This test showed no significant differences between groups or across trial blocks.

The significant effect of trial within the control group on the first testing day suggests that control animals increased HRA choices during the session while lesion animals did not. To follow-up on this effect, the first and last trial bins on testing day 1 were subjected to a paired samples t-test for each group separately (Figure 8C). This analysis showed that control rats improved significantly from trial bin 10 (*M* = 32.22, *SD* = 34.56) to trial bin 60 (*M* = 66.94, *SD* = 36.95), *t*_(8)_ = −4.12, *p* = 0.003 whereas lesion animals did not. In sum, ACC lesions did not affect the overall number of HRA choices in the face of fear but did stop rats from habituating to the high-fear/HRA.

#### Open field behavior

Following the aforementioned testing in Experiments 1 and 2, all rats were tested an 123 × 123 cm open field for 5 min to test for differences in anxiety or intrinsic activity levels. In Experiment 1, 9 rats with ACC lesions and 9 sham controls were tested. In Experiment 2, 12 lesion and 12 sham controls were tested. Dependent measures were time spent in center, path length, and running speed. No significant differences were observed in either experiment on any of the behavioral measures.

## Discussion

The primary purpose of this study was to examine the role of ACC in reward-related decisions involving different forms of costs, including two forms of physical effort as well as fear. Consistent with previous reports (Walton et al., 2002, 2003; Schweimer and Hauber, 2005; Rudebeck et al., 2006; Floresco and Ghods-Sharifi, 2007; Hauber and Sommer, 2009), we were able to show that rats with ACC lesions avoided a HRA that required the effort of climbing a wire mesh barrier. When effort involved pressing weighed levers to obtain reward, however, the picture was more complex. In Experiment 2, we found that two out of six of our lesioned animals stopped choosing the HRA while the remaining 4 animals performed no different than controls. A sensitive follow-up study (Experiment 3) using incremental increases in weights with no prior weight-training experience showed no differences between lesion and control groups. Based on the fact that 4 of our 6 lesioned animals in Experiment 2 were no different than controls and the lack of effect in Experiment 3, we conclude that the ACC is unlikely to be necessary for effort-reward decisions involving pressing weighted levers. Finally, when effort involved the courage to cross an exposed track to reach high reward, rats with ACC lesions were equally likely to enter the exposed arm. However, unlike controls, they failed to show any increase in high-fear/high-reward choice over time, suggesting a possible role for ACC in habituation or learning to overcome fear. Taken together, these results suggest that the role of rodent ACC in effort-reward decisions may be limited to only certain forms of physical effort.

We also report, for the first time, that ACC-lesioned rats in the ramp-climbing task were far more likely to turn back after reaching the barrier that sham controls. This effect was strongest in the first 10 trials, after which ACC lesioned rats shifted their initial choice more and more toward the LRA. This finding suggests that the ACC is not involved in the initial decision to turn into the HRA. Instead, its role seems to be limited to the point where rats are actually faced with a physical challenge and must decide whether to continue or turn around.

There are at least three different hypothesized functions of the ACC. In one view, the ACC encodes the amount of effort necessary to achieve a particular goal. It thus provides a signal that allows other systems to prepare for exerting the appropriate amount of effort (Walton et al., 2006). Lacking ACC input, it follows that animals cannot mobilize the necessary physical resources and hence will tend to choose easier options, if available, as has been amply demonstrated with rats on the ramp-climbing task (e.g., Walton et al., 2003). Consistent with this view, patients with damage to dorsal ACC show blunted affect when presented with mental or physical challenges (Critchley et al., 2003). A second view is that ACC encodes the net utility of a contemplated action, weighing both expected reward and effort cost (Cohen et al., 2007). This view has received support from both human fMRI as well as single-unit studies in primates and rats (Croxson et al., 2009; Kennerley et al., 2009; Prevost et al., 2010; Hillman and Bilkey, 2012). Both of these views suppose that the ACC is needed in order to make correct decisions. However, recent electrophysiological evidence has shown a lack of discriminative ACC activity when rats are making choices, although effort is clearly encoded in ACC just before it is exerted (Cowen et al., 2012). Another study has shown that ACC activity is necessary for maintenance of motor cortex activity related to a sustained motor response (Narayanan and Laubach, 2006). This electrophysiological evidence suggests the possibility that ACC is necessary for maintaining a course of action, especially when faced with interfering circumstances (Cowen et al., 2012). Our observation of rats choice behavior on the ramp-climbing task provide support for this view. Specifically, rats with ACC lesions were far more likely than controls to abort a HRA choice at the point where they actually encountered the ramp. This suggests that the ACC is needed when deciding to maintain a previously chosen course of action in the face of adversity.

Our results also provide insight into the task parameters that determine when effort-reward decision deficits will be manifest by rats with ACC lesions in the ramp-climbing task. While previous experiments have used discrete trials (e.g., Walton et al., 2002), we show that the same effect can be obtained when rats run continuously on a maze shaped like the Figure 8. In so doing, we were able to show that the effect is maintained across dozens of trials. However, subsequent testing using the same group of animals failed to show effects when the manner of testing was changed. Specifically, when we attempted to generate effort-discounting curves by incrementally increasing ramp height throughout a single session (Figure 4B), lesion animals choose the high-effort/HRA slightly less overall but there was no difference in the slope of the effort discounting curve between groups. Looking more carefully at the data, it is apparent that the slopes might have been different had sham animals performed better on the zero ramp condition. On their last day on the equate effort task, for example, the animals had near 100 percent HRA choices. It is possible that interference from the immediately preceding task (weight-lifting incremental) may have differentially interfered with the performance of sham rats. Another factor affecting these results is that rats in this experiment had several days of experience running the effort-reward task after surgery. Hence, it is possible that they may have learned to compensate for the lack of ACC input. This is consistent with previous evidence that experience on the ramp task can increase the degree to which a ACC-lesioned animal will choose the HRA (Walton et al., 2002; Experiment 2). A similar explanation may account for the lack of effect in our “challenge” test in which rats were re-trained without barriers and then suddenly presented with a very high barrier. However, in this case, it seems more likely that the effort outweighed the reward to such an extent that both lesion and control groups were equally inclined to avoid the high-effort/high-reward choice. Previous studies have shown that lesion animals can be enticed to climb a high barrier when the ratio of high to low reward is increased from 4:2 to 5:1 (Walton et al., 2002). Combined with our results, this suggests that ACC lesions affect decisions only in a narrow range where the reward outweighs the effort by only a slight margin. Our effort-discounting curve is consistent with this idea. Although not statistically significant, the greatest differences between groups were at midrange levels of effort and not at the extremes.

Given the well-substantiated role of the ACC in effort-reward decisions involving ramp climbing, the lack of effect when testing with weighed levers was surprising because both tasks involve exertion of physical effort. In Experiment 1, it seems quite likely that there was a lack of effect simply because the effort involved was too low. In other words, we encountered a ceiling effect. However, in Experiment 2, the effort was increased to such a high level that only 60% of our animals were able to reach training criteria. Despite this high level of effort, we failed to find a statistically significant effect of ACC lesions on decision performance. As previously noted, four of the six ACC lesioned animals who reach pre-training criterion on this task showed performance no different than controls while two animals showed a dramatic reduction in HRA choices. This binary outcome is quite different than the effects seen in the ramp climbing task, where most ACC lesioned animals showed some reduction in HRA climbs (compare Figures S1, S2). The fact that four lesioned animals performed the same as controls argues that ACC is not necessary for the effort-reward decision, itself, but may influence decisions in other ways, perhaps by making it harder for rats to physically depress the lever. Because the lever only triggered when fully depressed, lesioned animals who could not generate sufficient force quickly learned that pressing the high-reward lever was fruitless and correctly shifted their choices to the LRA. Given the binary outcomes for lesioned animals in Experiment 2, further testing would have required prohibitively large numbers of rats to obtain sufficient statistical power to definitely say one way or the other whether the ACC is necessary for weighted lever pressing. Instead, a follow-up study (Experiment 3) was run using an incremental increase in lever weight within a single session. This test, which was the first one run after surgery, avoids the potential confounds of task experience which may have clouded the results of incremental tests in Experiments 1 and 2. Further, by eliminating pre-training with weights, it increased the steepness of the effort discounting curves and potentially increased our ability to see effects due to lesions. Despite these conditions, we still failed to find any difference between lesion and control animals. Taken together, the results argue against a role for ACC in the decision phase of effort-reward tasks involving pressing weighted levers.

The different outcomes from our ramp-climbing and weighted lever experiments are puzzling. As noted above, both clearly involve physical effort. However, the ramp-climbing task presents a physically apparent impediment in the form of a looming ramp. The lever task, on the other hand, provides no visual cues as to the difficulty of a particular lever press. Instead, decisions must be based on past experience with each lever. Hence, it is possible that the ACC mediates effort-reward decisions in which effort is visually apparent but not in those that involve retrieving effort level from memory. However, the fact that lesioned rats initially choose the HRA but then turn back only after physically encountering the ramp argues against the idea that vision is a strong determinant of rats' choices in either task.

Another possibility is that ACC lesions cause impairments in motor control sufficient to impair climbing but not lever pressing. Both our own experiments and those of others demonstrate that rats with ACC lesions will choose to climb a high ramp to achieve high reward when the ramp height on both reward arms is equal. This finding certainly rules out gross motor deficits. However, it leaves open the possibility that ACC lesions cause subtle motor impairments that make it harder for lesioned animals to climb a wire mesh barrier and hence tip the balance toward the LRA. The fact that ACC lesioned animals in the ramp task initially entered the HRA but then turned back is consistent with the view. It is possible that rats only realized their physical limitations when actually touching the ramp and preparing to climb. Over several trials, they learned the high cost associated with climbing and eventually made decisions to avoid the HRA. This account could also explain why two of our ACC lesioned rats in the heavy-weight lever task seemed to have difficulty depressing the lever to its fully depressed position. In support of this hypothesis, the ACC has strong connections with adjacent motor control areas, notably primary motor cortex and secondary motor cortex (Heidbreder and Groenewegen, 2003; nomenclature from Paxinos and Watson, 2007). The ACC also has direct spinal cord projections (Gabbott et al., 2005). Further, damage that encompasses this region has been shown to cause deficits, albeit subtle ones, in the pellet reaching task (Whishaw et al., 1992).

Along similar lines, Hosokawa et al. (2013) have recently suggested that the ACC region lesioned in rodent barrier-climbing studies might be homologous to primate cingulate motor areas which are strongly modulated by the ongoing level of physical exertion. Hence, the observed behavioral deficits might be due to an inability to prepare for the necessary exertion of physical effort. This idea is certainly consistent with our observations, although further experiments, possibly involving high-speed video analysis or more sensitive measures of motor force, will be necessary to draw more definitive conclusions.

As mentioned previously, one fMRI study in humans has shown sub-genual ACC activity is correlated with courageous decisions (Nili et al., 2010). Our data suggest that, if such a region exists in rats, it is not located in the ACC. Or perhaps, as suggested by recent single-cell data, the ACC encodes both approach to reward and avoidance of aversive stimuli equally so that lesioning does not bias behavior in either direction (Amemori and Graybiel, 2012). In both of our courage experiments, rats with ACC lesions were no more likely to avoid the high-fear/HRA than controls. The results of our first courage experiment (Experiment 1) might be questioned based on the relatively low levels of fear involved (as evinced by the high number of entries into the exposed arm). However, in our second courage experiment (Experiment 2), both groups of animals were clearly inhibited from entering the high-fear/HRA and yet no group differences were observed. One caveat with these findings is that rats were trained and tested on the task post-surgery, whereas in the ramp and weight-lifting tasks, rats were pre-trained on the task before surgery and tested immediately after recovery. However, rats were never exposed to the open arm during pre-training, making specific post-lesion adjustment to the task an unlikely explanation for our lack of effect.

Given that lesions to medial prefrontal cortex just ventral to ACC are anxiolytic (Lacroix et al., 2000; Deacon et al., 2003; Shah and Treit, 2003), another possibility was that animals with ACC lesions could have been *more* likely to enter the high-fear/HRA. We found no evidence to support this view. We also failed to find evidence of reduced anxiety in the open field test, consistent with previous findings using other tests of intrinsic fear (Rudebeck et al., 2007). Interestingly, however, while normal animals gradually overcame their fear within the first session and entered the HRA more, animals with ACC lesions failed to show any such change. It is possible that this reflects better cost-benefit decision making in rats with an intact ACC in the same way that the ramp results suggest control animals are better able to reason about effort and reward. However, the pattern of results across time is completely different. In the ramp-climbing results, both our own and those in the original Walton studies (Walton et al., 2002, 2003), rats with ACC lesions show an immediate reduction in HRA choices. In the courage task results, on the other hand, there is no initial difference between groups. In sum, the ramp task shows that ACC rats have an immediate impairment in decision making while the courage task shows an impairment in learning.

In conclusion, our results provide support for the idea that different regions of frontal cortex mediate different forms of cost-benefit decision making, as has previously been suggested (Rudebeck et al., 2006; Walton et al., 2006; Floresco et al., 2008; Prevost et al., 2010). The ACC clearly plays a role in effort-reward decisions involving ramp climbing (e.g., Walton et al., 2003) and possibly pressing levers multiple times [but see Schweimer and Hauber (2005), Walton et al. (2009)]. Our results, however, suggest that the problem may be in following through on the effortful climb once chosen and not in the actual effort-reward decision. Our results also show that when effort involves pressing weighted levers, the ACC plays, at most, only a limited role. Similarly, the choice to wait for a large reward clearly does not require the ACC, depending instead upon nucleus accumbens and orbitofrontal cortex (Cardinal et al., 2001; Rudebeck et al., 2006). Finally, based on our results, we can now conclude that, at least in rats, ACC is not required in cost-benefit decisions involving fear. Exactly which part of the rodent brain enables pursuit of goals in the face of fear remains an open question.

### Conflict of interest statement

The authors declare that the research was conducted in the absence of any commercial or financial relationships that could be construed as a potential conflict of interest.

## References

[B1] Amemori K. Graybiel A. M. (2012). Localized microstimulation of primate pregenual cingulate cortex induces negative decision-making. Nat. Neurosci. 15, 776–785 10.1038/nn.3088 22484571 PMC3369110

[B2] Botvinick M. M. Braver T. S. Barch D. M. Carter C. S. Cohen J. D. (2001). Conflict monitoring and cognitive control. Psychol. Rev. 108, 624–652 10.1037/0033-295X.108.3.624 11488380

[B3] Botvinick M. M. Huffstetler S. McGuire J. T. (2009). Effort discounting in human nucleus accumbens. Cogn. Affect. Behav. Neurosci. 9, 16–27 10.3758/CABN.9.1.16 19246324 PMC2744387

[B4] Cardinal R. N. Pennicott D. R. Sugathapala C. L. Robbins T. W. Everitt B. J. (2001). Impulsive choice induced in rats by lesions of the nucleus accumbens core. Science 292, 2499–2501 10.1126/science.1060818 11375482

[B5] Cohen J. D. McClure S. M. Yu A. J. (2007). Should I stay or should I go? How the human brain manages the trade-off between exploitation and exploration. Philos. Trans. R. Soc. Lond. B Biol. Sci. 362, 933–942 10.1098/rstb.2007.2098 17395573 PMC2430007

[B6] Cowen S. L. Davis G. A. Nitz D. A. (2012). Anterior cingulate neurons in the rat map anticipated effort and reward to their associated action sequences. J. Neurophysiol. 107, 2393–2407 10.1152/jn.01012.2011 22323629

[B7] Critchley H. D. Mathias C. J. Josephs O. O'Doherty J. Zanini S. Dewar B. K. (2003). Human cingulate cortex and autonomic control: converging neuroimaging and clinical evidence. Brain 126, 2139–2152 10.1093/brain/awg216 12821513

[B8] Croxson P. L. Walton M. E. O'Reilly J. X. Behrens T. E. Rushworth M. F. (2009). Effort-based cost-benefit valuation and the human brain. J. Neurosci. 29, 4531–4541 10.1523/JNEUROSCI.4515-08.2009 19357278 PMC2954048

[B9] Deacon R. M. Penny C. Rawlins J. N. (2003). Effects of medial prefrontal cortex cytotoxic lesions in mice. Behav. Brain Res. 139, 139–155 10.1016/S0166-4328(02)00225-5 12642185

[B10] Euston D. R. Gruber A. J. McNaughton B. L. (2012). The role of medial prefrontal cortex in memory and decision making. Neuron 76, 1057–1070 10.1016/j.neuron.2012.12.002 23259943 PMC3562704

[B11] Floresco S. B. Ghods-Sharifi S. (2007). Amygdala-prefrontal cortical circuitry regulates effort-based decision making. Cereb. Cortex 17, 251–260 10.1093/cercor/bhj143 16495432

[B12] Floresco S. B. St. Onge J. R. Ghods-Sharifi S. Winstanley C. A. (2008). Cortico-limbic-striatal circuits subserving different forms of cost-benefit decision making. Cogn. Affect. Behav. Neurosci. 8, 375–389 10.3758/CABN.8.4.375 19033236

[B13] Gabbott P. L. Warner T. A. Jays P. R. Salway P. Busby S. J. (2005). Prefrontal cortex in the rat: projections to subcortical autonomic, motor, and limbic centers. J. Comp. Neurol. 492, 145–177 10.1002/cne.20738 16196030

[B14] Hauber W. Sommer S. (2009). Prefrontostriatal Circuitry Regulates Effort-Related Decision Making. Cereb. Cortex 19, 2240–2247 10.1093/cercor/bhn241 19131436

[B15] Heidbreder C. A. Groenewegen H. J. (2003). The medial prefrontal cortex in the rat: evidence for a dorso-ventral distinction based upon functional and anatomical characteristics. Neurosci. Biobehav. Rev. 27, 555–579 10.1016/j.neubiorev.2003.09.003 14599436

[B16] Hillman K. L. Bilkey D. K. (2010). Neurons in the rat anterior cingulate cortex dynamically encode cost-benefit in a spatial decision-making task. J. Neurosci. 30, 7705–7713 10.1523/JNEUROSCI.1273-10.2010 20519545 PMC6632387

[B17] Hillman K. L. Bilkey D. K. (2012). Neural encoding of competitive effort in the anterior cingulate cortex. Nat. Neurosci. 15, 1290–1297 10.1038/nn.3187 22885851

[B18] Hogg S. (1996). A review of the validity and variability of the elevated plus-maze as an animal model of anxiety. Pharmacol. Biochem. Behav. 54, 21–30 10.1016/0091-3057(95)02126-4 8728535

[B19] Holroyd C. B. Coles M. G. (2002). The neural basis of human error processing: reinforcement learning, dopamine, and the error-related negativity. Psychol. Rev. 109, 679–709 10.1037/0033-295X.109.4.679 12374324

[B20] Hosokawa T. Kennerley S. W. Sloan J. Wallis J. D. (2013). Single-neuron mechanisms underlying cost-benefit analysis in frontal cortex. J. Neurosci. 33, 17385–17397 10.1523/JNEUROSCI.2221-13.2013 24174671 PMC3812506

[B21] Kennerley S. W. Dahmubed A. F. Lara A. H. Wallis J. D. (2009). Neurons in the frontal lobe encode the value of multiple decision variables. J. Cogn. Neurosci. 21, 1162–1178 10.1162/jocn.2009.21100 18752411 PMC2715848

[B22] Kurniawan I. T. Guitart-Masip M. Dayan P. Dolan R. J. (2013). Effort and valuation in the brain: the effects of anticipation and execution. J. Neurosci. 33, 6160–6169 10.1523/JNEUROSCI.4777-12.2013 23554497 PMC3639311

[B23] Lacroix L. Spinelli S. Heidbreder C. A. Feldon J. (2000). Differential role of the medial and lateral prefrontal cortices in fear and anxiety. Behav. Neurosci. 114, 1119–1130 10.1037/0735-7044.114.6.1119 11142644

[B24] McDonald R. J. King A. L. Foong N. Rizos Z. Hong N. S. (2008). Neurotoxic lesions of the medial prefrontal cortex or medial striatum impair multiple-location place learning in the water task: evidence for neural structures with complementary roles in behavioural flexibility. Exp. Brain Res. 187, 419–427 10.1007/s00221-008-1314-z 18330551

[B25] Narayanan N. S. Laubach M. (2006). Top-down control of motor cortex ensembles by dorsomedial prefrontal cortex. Neuron 52, 921–931 10.1016/j.neuron.2006.10.021 17145511 PMC3995137

[B26] Nili U. Goldberg H. Weizman A. Dudai Y. (2010). Fear thou not: activity of frontal and temporal circuits in moments of real-life courage. Neuron 66, 949–962 10.1016/j.neuron.2010.06.009 20620879

[B27] Passingham R. E. Bengtsson S. L. Lau H. C. (2010). Medial frontal cortex: from self-generated action to reflection on one's own performance. Trends Cogn. Sci. 14, 16–21 10.1016/j.tics.2009.11.001 19969501 PMC2806969

[B28] Paxinos G. Watson C. (2007). The Rat Brain, 6th Edn. San Diego, CA: Academic Press

[B29] Prevost C. Pessiglione M. Metereau E. Clery-Melin M. L. Dreher J. C. (2010). Separate valuation subsystems for delay and effort decision costs. J. Neurosci. 30, 14080–14090 10.1523/JNEUROSCI.2752-10.2010 20962229 PMC6634773

[B30] Rudebeck P. H. Walton M. E. Millette B. H. Shirley E. Rushworth M. F. Bannerman D. M. (2007). Distinct contributions of frontal areas to emotion and social behaviour in the rat. Eur. J. Neurosci. 26, 2315–2326 10.1111/j.1460-9568.2007.05844.x 17927774 PMC2228395

[B31] Rudebeck P. H. Walton M. E. Smyth A. N. Bannerman D. M. Rushworth M. F. (2006). Separate neural pathways process different decision costs. Nat. Neurosci. 9, 1161–1168 10.1038/nn1756 16921368

[B32] Rushworth M. F. (2008). Intention, choice, and the medial frontal cortex. Ann. N.Y. Acad. Sci. 1124, 181–207 10.1196/annals.1440.014 18400931

[B33] Salamone J. D. Cousins M. S. Bucher S. (1994). Anhedonia or anergia? Effects of haloperidol and nucleus accumbens dopamine depletion on instrumental response selection in a T-maze cost/benefit procedure. Behav. Brain Res. 65, 221–229 10.1016/0166-4328(94)90108-2 7718155

[B34] Schweimer J. Hauber W. (2005). Involvement of the rat anterior cingulate cortex in control of instrumental responses guided by reward expectancy. Learn. Mem. 12, 334–342 10.1101/lm.90605 15930509 PMC1142463

[B35] Shackman A. J. Salomons T. V. Slagter H. A. Fox A. S. Winter J. J. Davidson R. J. (2011). The integration of negative affect, pain and cognitive control in the cingulate cortex. Nat. Rev. Neurosci. 12, 154–167 10.1038/nrn2994 21331082 PMC3044650

[B36] Shah A. A. Treit D. (2003). Excitotoxic lesions of the medial prefrontal cortex attenuate fear responses in the elevated-plus maze, social interaction and shock probe burying tests. Brain Res. 969, 183–194 10.1016/S0006-8993(03)02299-6 12676379

[B37] Solinsky C. Kirby B. P. (2013). Medial prefrontal cortex lesions in mice do not impair effort-based decision making. Neuropharmacology 65, 223–231 10.1016/j.neuropharm.2012.10.005 23092919

[B38] Walton M. E. Bannerman D. M. Alterescu K. Rushworth M. F. (2003). Functional specialization within medial frontal cortex of the anterior cingulate for evaluating effort-related decisions. J. Neurosci. 23, 6475–6479 12878688 10.1523/JNEUROSCI.23-16-06475.2003 PMC6740644

[B39] Walton M. E. Bannerman D. M. Rushworth M. F. (2002). The role of rat medial frontal cortex in effort-based decision making. J. Neurosci. 22, 10996–11003 12486195 10.1523/JNEUROSCI.22-24-10996.2002 PMC6758435

[B40] Walton M. E. Groves J. Jennings K. A. Croxson P. L. Sharp T. Rushworth M. F. (2009). Comparing the role of the anterior cingulate cortex and 6-hydroxydopamine nucleus accumbens lesions on operant effort-based decision making. Eur. J. Neurosci. 29, 1678–1691 10.1111/j.1460-9568.2009.06726.x 19385990 PMC2954046

[B41] Walton M. E. Kennerley S. W. Bannerman D. M. Phillips P. E. Rushworth M. F. (2006). Weighing up the benefits of work: behavioral and neural analyses of effort-related decision making. Neural Netw. 19, 1302–1314 10.1016/j.neunet.2006.03.005 16949252 PMC2519033

[B42] Whishaw I. Q. Pellis S. M. Gorny B. P. (1992). Medial frontal cortex lesions impair the aiming component of rat reaching. Behav. Brain Res. 50, 93–104 10.1016/S0166-4328(05)80291-8 1449652

