# Supplementary material for: Not all effort is equal: the role of the anterior cingulate cortex in different forms of effort-reward decisions
Source: Front Behav Neurosci. 2014 Jan 28;8:12. doi: 10.3389/fnbeh.2014.00012 (PMC3904092; doi:10.3389/fnbeh.2014.00012)
Supplement: Supplementary file 1 [file Presentation1.PDF]

Supplementary Material

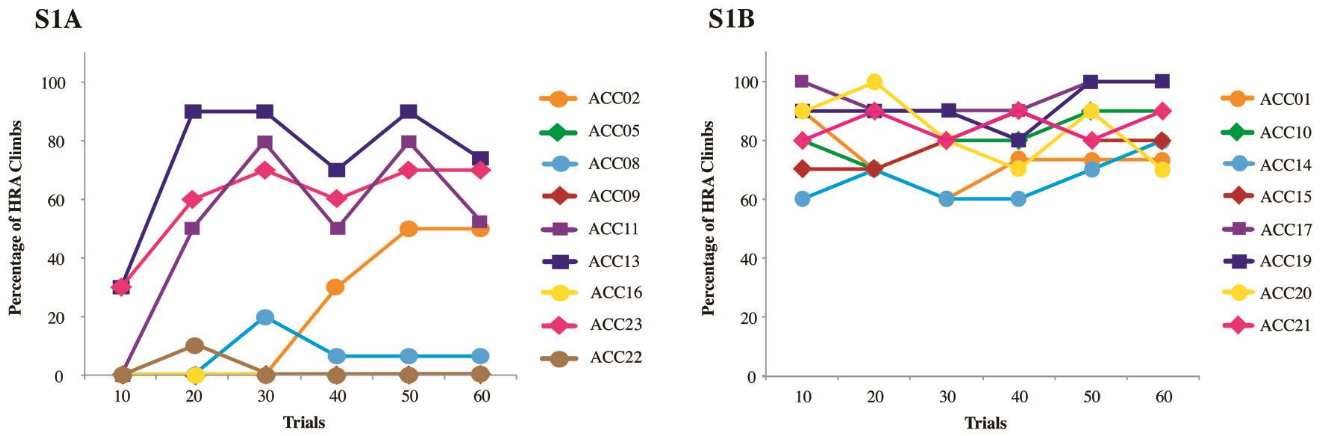

Figure S1 | Performance of Individual Rats on Experiment 1 Ramp Climbing Task, Testing Day 1 Post-Surgery. Graphs shown the percentage of high-reward arm successful climbs for (A) rats with ACC lesions and (B) sham controls. Data is the same as shown in Figure 4A for Testing Day 1.

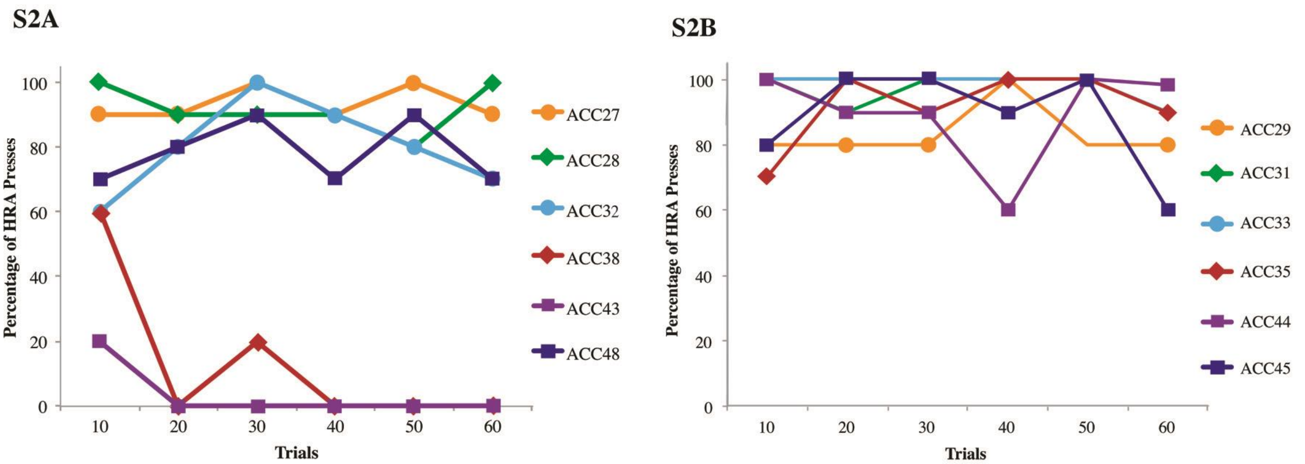

Figure S2 | Performance of Individual Rats on Experiment 2 Weight-Lifting Task, Testing Day 1 Post-Surgery. Graphs shown the percentage of high-reward arm successful lever presses for (A) rats with ACC lesions and (B) sham controls. Data is the same as shown in Figure 6B for Testing Day 1.
